# Efficacy of direct oral anticoagulant for renal infarction due to pulmonary vein stump thrombosis after left pneumonectomy

**DOI:** 10.1186/s40792-022-01574-8

**Published:** 2022-12-02

**Authors:** Yoshihito Iijima, Shun Iwai, Nozomu Motono, Hidetaka Uramoto

**Affiliations:** Department of Thoracic Surgery, Kanazawa Medical University, 1-1 Daigaku, Kahoku-Gun, Uchinada-Machi, Ishikawa 920-0293 Japan

**Keywords:** Lung cancer, Pneumonectomy, Pulmonary vein stump thrombosis, Renal infarction, Direct oral anticoagulant

## Abstract

**Background:**

Thrombosis of the pulmonary vein stump is a common complication after a left upper lobectomy and can be a source of embolism in various organs, such as the kidneys. A renal infarction, on the other hand, is a rare and often lethal condition that is usually diagnosed postmortem. Here, we present a case of renal infarction associated with pulmonary vein stump thrombosis after a left pneumonectomy, which was treated successfully with edoxaban.

**Case presentation:**

A 73-year-old man was diagnosed with squamous cell lung cancer (cT1miN0M0 stage IA1). Due to pneumoconiosis, extensive lymph node infiltration into the pulmonary artery was noted and necessitated an open thoracotomy. Ultimately, a left pneumonectomy was performed. Atrial fibrillation occurred on postoperative day 3, and the patient complained of left-sided abdominal pain. Contrast-enhanced computed tomography revealed a left upper pulmonary vein thrombosis and a left renal infarction. Anticoagulant therapy was immediately initiated with heparin and warfarin. On postoperative day 13, warfarin was replaced with the direct oral anticoagulant edoxaban since the patient’s compliance and drug response to warfarin were poor. On postoperative day 19, contrast-enhanced computed tomography revealed a reduction in pulmonary vein stump thrombosis and improvement in renal infarction. Subsequently, the patient was discharged. Three months post-surgery, no infarct lesions or reduced renal function was observed on imaging.

**Conclusions:**

The direct oral anticoagulant edoxaban could be effective in preventing recurrence or exacerbation of pulmonary vein thrombosis associated with renal infarction without bleeding complications.

## Background

Renal infarction (RI) after lung resection is rare and sometimes lethal. In contrast, the incidence of pulmonary vein stump thrombosis (PVST) after left upper lobectomy is as high as 13.5% [1]. It is a source of embolism in various organs, including the kidneys and the brain [2–4]. PVST is present in 33% of reported RI after lung resection, and almost all patients receive anticoagulant therapy with heparin, warfarin, or dipyridamole [5]. However, no standard guidelines exist regarding PVST anticoagulant treatment.

Here, we report a case of PVST associated with RI after a left pneumonectomy for lung cancer, effectively treated with the anticoagulant edoxaban.

## Case presentation

**Fig. 1:**
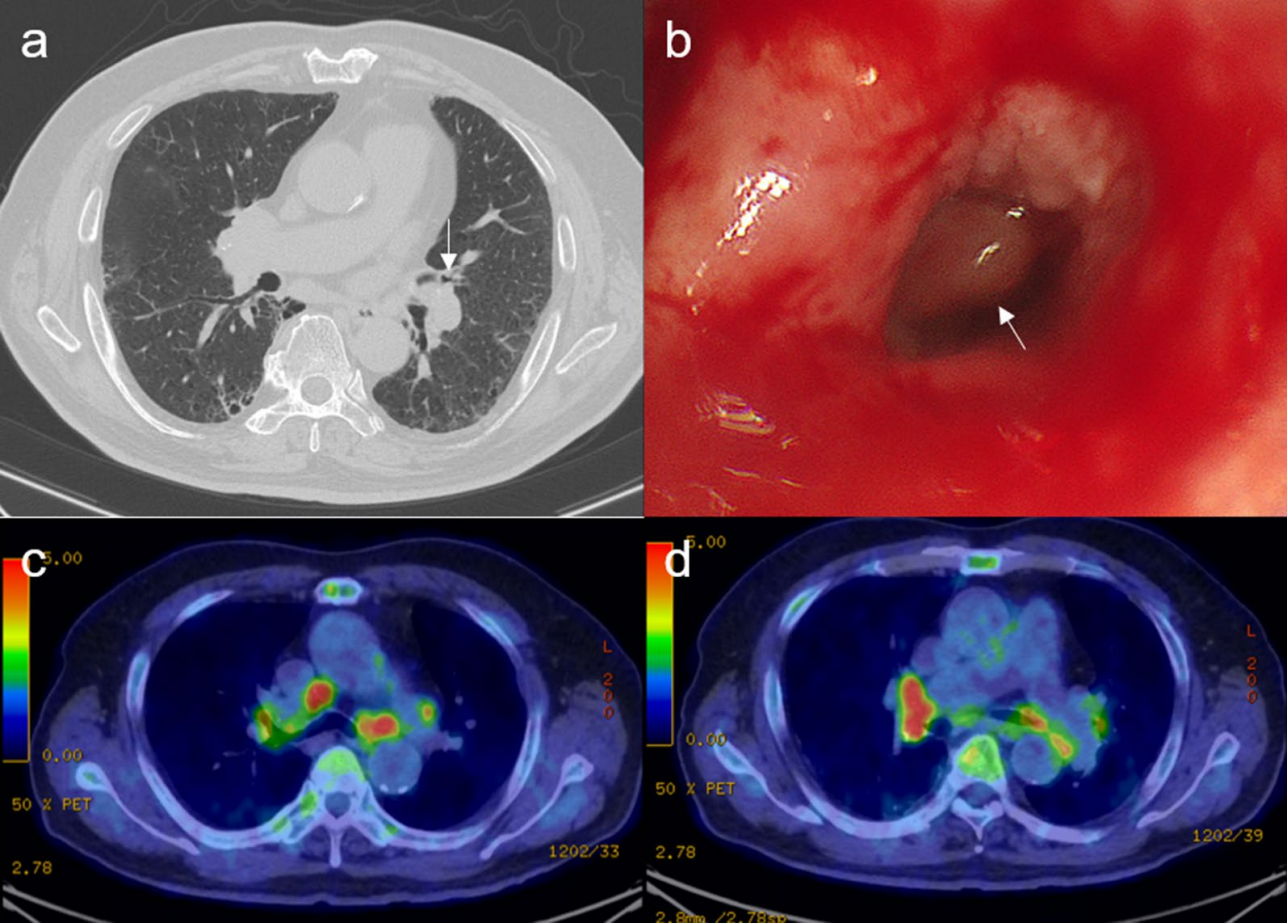
Preoperative findings. **a** Computed tomography shows a polypoid nodular shadow in the left superior lingual bronchus (white arrow) without lymphadenopathy. **b** Bronchoscopy shows a polypoid nodule in the left superior lingual bronchus (white arrow). **c**, **d** Accumulation of 2-deoxy-2-(18F)-fluorodeoxyglucose up to the maximal standardized uptake value of 6.0 is observed in the bilateral hilar and mediastinal lymph nodes

A 73-year-old man with a history of hypertension, diabetes, pneumoconiosis, and chronic obstructive lung disease was diagnosed with squamous cell lung cancer, staged as cT1miN0M0 and stage IA1. The patient was a plumber and heavy smoker with a Brinkman index of 2700. Computed tomography (CT) showed a polypoid nodular shadow in the left superior lingual bronchus (Fig. 1a, b). Nuclear tracer accumulation was visualized in a solitary pulmonary nodule on 2-deoxy-2-(^18^F)-fluorodeoxyglucose (FDG) positron emission tomography. Additionally, FDG accumulation up to the maximum standardized uptake value of 6 was observed in the bilateral hilar and mediastinal lymph nodes (Fig. 1c, d). Endobronchial treatment, such as photodynamic therapy, was considered, although the patient preferred surgical resection.

Initially, we attempted a left lingual segmentectomy using video-assisted thoracoscopic surgery. However, as a consequence of pneumoconiosis, we noted intrapulmonary lymph node infiltration into the pulmonary artery. Consequently, we were compelled to convert to open thoracotomy. The left main pulmonary artery was clamped, and lymph node dissection was attempted. There was, however, difficulty in dissecting the lymph nodes that had been extensively involved. Furthermore, we were unable to perform a pulmonary angioplasty and thus performed a left pneumonectomy. The duration of the surgery was 138 min, and the amount of blood loss was approximately 90 g.

**Fig. 2:**
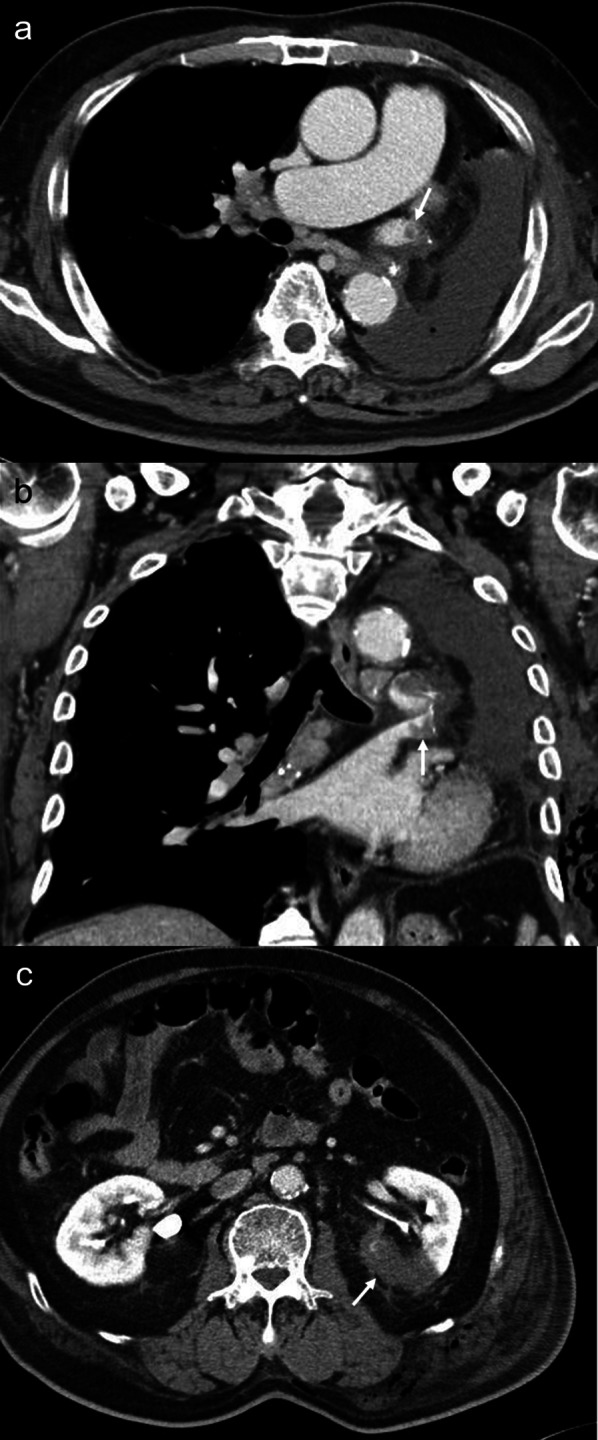
Contrast-enhanced computed tomography reveals a thrombus in the left upper pulmonary vein stump: **a** coronal view, **b** horizontal view, and **c** partial defect of the left kidney on postoperative day 4

**Fig. 3:**
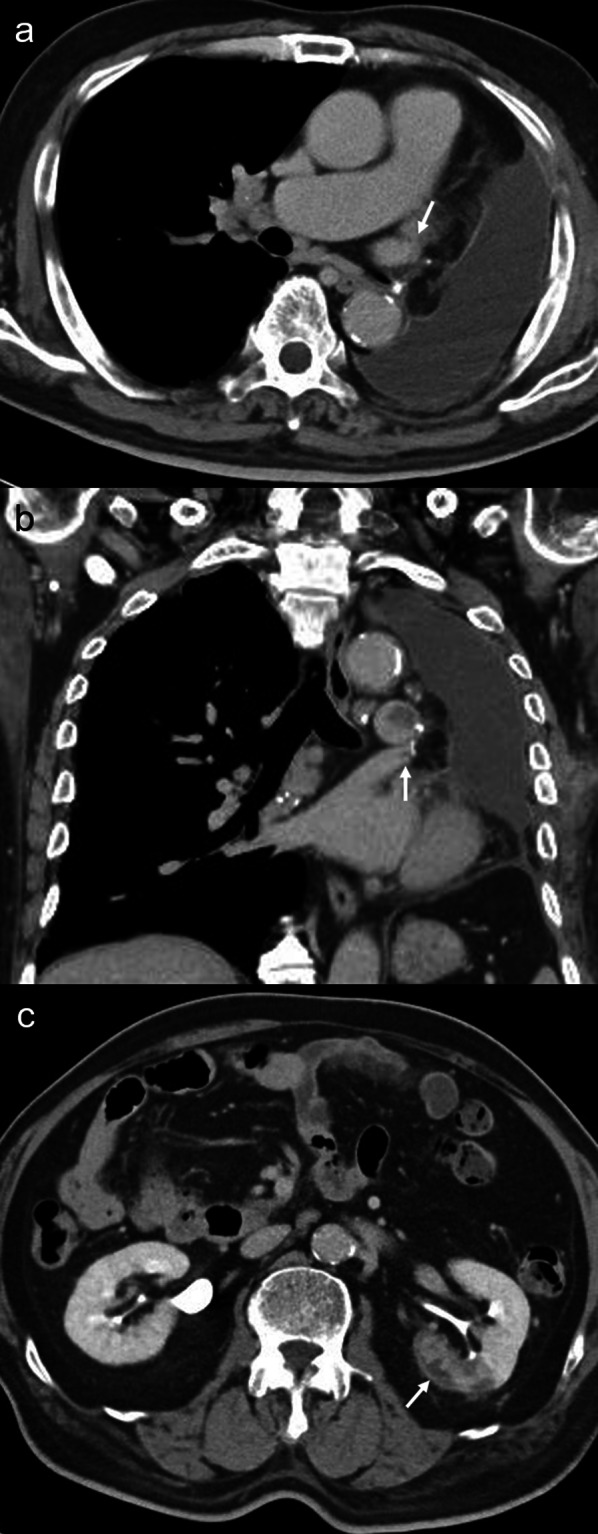
A contrast-enhanced computed tomography on postoperative day 19 reveals a reduction of the thrombus in the left upper pulmonary vein stump: **a** coronal view, **b** horizontal view, and **c** recovery of the partial defect of the left kidney

The chest drainage tube was removed on postoperative day (POD) 1. Atrial fibrillation (AF) occurred on POD 3, but sinus rhythm was re-established with verapamil. On POD 4, the patient began complaining of left abdominal pain. He experienced rapid increases in lactate dehydrogenase (LDH) and leukocyte levels, reaching 1129 IU/L and 13,100 cells/mcl, respectively. A thoracoabdominal contrast-enhanced CT (CE-CT) revealed left upper PVST and left RI (Fig. 2). To prevent progression of RI, 200 IU/kg of heparin was administered immediately. On POD 5, oral warfarin was initiated. Unfortunately, the patient exhibited poor compliance and response to warfarin, as his prothrombin time was not prolonged sufficiently. Therefore, we decided to change to edoxaban as his renal function was maintained on POD 13. Heparin was discontinued on POD 15. A thoracoabdominal CE-CT performed on POD 19 revealed a reduction in PVST and an improvement of RI (Fig. 3). The patient was discharged on POD 20. Infarct lesions or worsening renal function were not observed 3 months after the operation. Administration of edoxaban was ongoing at the time.

## Discussion

RI is evidently an extremely rare disease, with an incidence rate of 0.007% [6]. Since only 1% of RIs found at autopsy are diagnosed antemortem [7], many of them are overlooked, and the exact number is still unknown. In many instances, the symptoms are nonspecific and may be mistaken for those caused by renal pyelonephritis, renal colic, acute mesenteric events, or urinary tract infections, which can cause delays in diagnosis and treatment [5, 6]. Previous studies have reported elevations in white blood cell count, LDH, C-reactive protein, and creatinine in blood investigations, as well as hematuria in urinalysis [5].

Thrombus formation could arise from the stump of the left upper pulmonary vein as it tends to be anatomically long. A thrombus here might develop because of turbulent flow or stasis of blood in the stump [1]. There have been only 13 reports of RI after surgery for lung cancer (Table 1) [4, 5, 8–17], including our case, and they included left lung cancer with isolation of the left superior pulmonary vein. In these cases, the median time to RI onset was POD 4 (interquartile range, POD 3–12). Moreover, PVST was noted in seven cases (53.8%). Synchronous non-renal organ infarction was reported in four cases. Two patients had AF before RI onset. In 11 patients, heparin with or without warfarin was administered as an anticoagulant therapy. There is no consistent trend in the laterality and extent of RI. Small or unilateral infarcts may not show renal dysfunction due to contralateral renal compensation [16]. Elevated LDH was reported in eight cases. LDH elevation associated with flank lateral abdominal pain and low back pain is important as this finding is suggestive of RI [14–16].

**Table 1:** Reported cases of renal infarction after pulmonary resection

Case	Age	Sex	Procedure	PVST	Onset	Symptom		Postoperative Af	DM	HT	History of organ infarction	Laterality of RI	Extent of RI
						Abdominal pain	Others						
1	80	F	LUL	−	5 days	−	−	−	−	−	−	R	Partial
2	71	F	LUL	+	12 days	Abdominal pain	ND	+	−	+	−	B	Multiple
3	53	F	LUL	−	3 days	Left flank pain	ND	−	−	−	−	L	Filling defect in the left RA
4	52	F	LUL	−	6 h	Right upper abdominal pain	Fever	−	−	−	−	R	Partial, cortex
10	61	F	LUL	+	4 days	Right abdominal pain	ND	ND	ND	ND	ND	R	Wedge shaped
5	76	M	LUL	−	15 days	Left lower abdominal pain	Vomiting, diarrhea	ND	−	−	−	R	Extensive
6	76	M	LUL	+	13 months	Right flank pain	ND	ND	ND	ND	ND	L	Large, wedge shaped
7	70	M	LUL	−	4 days	Abdominal pain	Fever	−	+	+	−	R	ND (flip-flop enhancement)
8	68	M	LUL	−	4 days	Left back pain	Nausea, vomiting	−	−	+	CI	L	ND (poor contrast)
9	68	M	LUL	+	2 days	Left flank pain	ND	−	−	−	−	L	Large, excluding upper pole
11	43	M	LUL	+	14 days	Left flank pain	Fever	−	−	−	−	L	Large, wedge shaped
12	70	M	LUL	+	12 days	−	−	−	−	+	AMI	R	Partial, upper pole
13	73	M	LPn	+	4 days	Left abdominal pain	−	+	+	+	−	L	Partial

Case	Preoperative CRE (mg/dL)	Post-RI peak of CRE (mg/dL)	Elevation of LDH (peak; IU/L)	Other organ infarction	Anticoagulant therapy			References
					Heparin	Warfarin	Other	
1	ND	ND	+ (670)	−	+	-	−	[5]
2	0.63	ND	ND	Brain, spleen, SMA,	+	+	−	[8]
3	0.71	0.83	+ (370)	−	+	+	−	[9]
4	WNL	ND	+ (1764)	−	-	-	Dipyridamole	[10]
10	ND	ND	ND	−	+	+	−	[11]
5	ND	1.44	ND	SMA	+	+	−	[12]
6	ND	ND	ND	−	+	+	−	[13]
7	0.9	Mild elevation	+ (1402)	−	−	−	−	[14]
8	WNL	ND	+	RCIA	+	+	−	[15]
9	WNL	ND	+	IMA?	+	+	−	[16]
11	ND	0.95	+ (1530)	−	+	+	−	[17]
12	WNL	ND	ND	−	+	−	Rivaroxaban	[4]
13	0.64	0.9	+ (1129)	−	+	+	Edoxaban	Our case

*PVST* pulmonary vein stump thrombosis, *Af* atrial fibrillation, *DM* diabetes mellitus, *HT* hypertension, *RI* renal infarction, *CRE* creatinine, *LDH* lactate dehydrogenase, *F* female, *M* male, *LUL* left upper lobectomy, *LPn* left pneumonectomy, *ND* not described, *CI* cerebral infarction, *AMI* acute myocardial infarction, *R* right, *B* bilateral, *L* left, *RA* renal artery, *WNL* within normal limit, *SMA* superior mesenteric artery, *RCIA* right common iliac artery, *IMA* inferior mesenteric artery

As of yet, RI risk stratification has not been established. However, it has been reported that 44.7% of patients with RI have AF or intracardiac thrombosis [18], and the risk of RI is increased in patients with a history of cerebral infarction [19]. Thus, risk factors for RI can possibly be identified by investigating the risk factors involved in cardiogenic cerebral embolism.

Treatments for RI include transvenous thrombolysis, anticoagulant therapy, and percutaneous transluminal angioplasty (PTA) via a catheter. Adaptation to PTA generally takes approximately 6–12 h. However, it has been reported that, beyond 24 h after onset, there is no significant difference in the response rate between intravenous thrombolysis combined with anticoagulant therapy and anticoagulant therapy alone [20, 21]. Typically, it takes 43 h between the onset of symptoms and diagnosis or treatment [20], and anticoagulant therapy is used in many cases. While anticoagulant therapy can be initiated, there are no standard guidelines for its duration. Due to the rare frequency of RI cases, further investigation is required to determine the best treatment for these conditions and the effectiveness of anticoagulant therapy for PVST. Few reports have described the use of direct oral anticoagulants (DOACs) for RI after PVST. Despite this, we believe that the use of DOACs will increase in the future because they are relatively safe. Song et al. reduced the dose of rivaroxaban after confirming the resolution of PVST by CE-CT 1 year post-surgery [4]. The study further reported that rivaroxaban was completely discontinued 2 years post-surgery, after the absence of PVST recurrence was confirmed on CE-CT. Similarly, our case also required strict long-term follow-up.

## Conclusions

We presented a rare case of RI that developed shortly after left pneumonectomy. Following surgery, the patient developed AF, and a CE-CT imaging investigation detected PVST. With the use of edoxaban as an anticoagulant therapy, the patient experienced a favorable clinical outcome but will require a strict long-term follow-up.

## Data Availability

All data generated or analyzed during this study are included in this published article.

## Abbreviations

RI: Renal infarction
PVST: Pulmonary vein stump thrombosis
FDG: 2-Deoxy-2-(18F)-fluorodeoxyglucose
POD: Postoperative day
AF: Atrial fibrillation
LDH: Lactate dehydrogenase
CE-CT: Contrast-enhanced computed tomography
DOAC: Direct oral anticoagulants
